# Supplementary material for: Tracing the path of 37,050 studies into practice across 18 specialties of the 2.4 million published between 2011 and 2020
Source: eLife. 2023 Feb 28;12:e82498. doi: 10.7554/eLife.82498 (PMC10115455; doi:10.7554/eLife.82498)
Supplement: Supplementary file 1. [file elife-82498-supp1.docx]

| **Specialty** | **Journal** | **Median Years to Citation** | **Fraction of Articles Cited** | **5-Year Impact Factor** |
| --- | --- | --- | --- | --- |
| **anesthesiology** | Br J Anaesth | 3 | 0.009 | 9.097 |
|  | Anesthesiology | 5 | 0.011 | 8.139 |
|  | Anaesthesia | 3 | 0.005 | 6.211 |
|  | Reg Anesth Pain Med | 4 | 0.017 | 6.077 |
|  | Eur J Anaesthesiol | 2 | 0.005 | 4.941 |
|  | Anesth Analg | 3 | 0.013 | 4.935 |
|  | Can J Anaesth | 4 | 0.009 | 4.362 |
|  | J Clin Anesth | 4 | 0.006 | 4.067 |
|  | Best Pract Res Clin Anaesthesiol | 5 | 0.01 | 3.397 |
|  | Curr Opin Anaesthesiol | 1 | 0.015 | 2.824 |
|  | BMC Anesthesiol | 4 | 0.023 | 2.477 |
|  | Acta Anaesthesiol Scand | 2.5 | 0.007 | 2.469 |
|  | J Anesth | 1 | 0.005 | 1.975 |
|  | Rev Bras Anestesiol | 7.5 | 0.01 | 1.183 |
|  | Anaesthesist | 2 | 0.004 | 1.094 |
| **cardiac & cardiovascular systems** | Eur Heart J | 1 | 0.022 | 29.729 |
|  | Nat Rev Cardiol | 1 | 0.015 | 27 |
|  | J Am Coll Cardiol | 2 | 0.044 | 22.758 |
|  | Eur J Heart Fail | 3 | 0.012 | 12.768 |
|  | JACC Heart Fail | 1 | 0.047 | 11.282 |
|  | JACC Cardiovasc Interv | 1 | 0.035 | 10.731 |
|  | J Am Soc Echocardiogr | 2 | 0.019 | 9.01 |
|  | Cardiovasc Res | 15 | 0.002 | 8.861 |
|  | Circ Heart Fail | 2 | 0.04 | 8.509 |
|  | Prog Cardiovasc Dis | 5.5 | 0.009 | 7.107 |
|  | Circ Cardiovasc Interv | 1.5 | 0.032 | 6.891 |
|  | Circ Arrhythm Electrophysiol | 1 | 0.047 | 6.6 |
|  | Eur J Prev Cardiol | 1 | 0.01 | 6.361 |
|  | Heart | 2 | 0.014 | 6.308 |
|  | Heart Rhythm | 1 | 0.037 | 6.209 |
|  | Circ Cardiovasc Qual Outcomes | 1 | 0.034 | 6.168 |
|  | J Am Heart Assoc | 1 | 0.023 | 6.155 |
|  | Curr Probl Cardiol | 1 | 0.077 | 5.492 |
|  | Europace | 1 | 0.018 | 5.274 |
|  | Can J Cardiol | 3 | 0.009 | 5.226 |
|  | J Card Fail | 5 | 0.024 | 5.208 |
|  | Heart Fail Rev | 1 | 0.022 | 5.201 |
|  | Clin Res Cardiol | 6 | 0.008 | 4.901 |
|  | Rev Esp Cardiol | 10 | 0.006 | 4.831 |
|  | Am Heart J | 5 | 0.006 | 4.569 |
|  | EuroIntervention | 1.5 | 0.005 | 4.439 |
|  | Int J Cardiol | 2 | 0.004 | 4.24 |
|  | ESC Heart Fail | 2 | 0.03 | 4.072 |
|  | Glob Heart | 1 | 0.019 | 4.028 |
|  | Eur Heart J Acute Cardiovasc Care | 0 | 0.021 | 3.702 |
|  | Circ J | 3 | 0.006 | 3.152 |
|  | Am J Cardiol | 3 | 0.011 | 3.133 |
|  | Arch Cardiovasc Dis | 1 | 0.007 | 3.093 |
|  | Curr Cardiol Rep | 2 | 0.005 | 2.966 |
|  | Clin Cardiol | 7 | 0.005 | 2.88 |
|  | J Cardiol | 0 | 0.004 | 2.742 |
|  | Heart Lung Circ | 1 | 0.006 | 2.682 |
|  | Curr Opin Cardiol | 2 | 0.011 | 2.628 |
|  | BMC Cardiovasc Disord | 4 | 0.006 | 2.613 |
|  | Cardiovasc Ultrasound | 4 | 0.007 | 2.574 |
|  | Heart Fail Clin | 1.5 | 0.011 | 2.508 |
|  | Cardiol Clin | 2.5 | 0.009 | 2.481 |
|  | Catheter Cardiovasc Interv | 3 | 0.007 | 2.461 |
|  | Cardiol Rev | 7.5 | 0.025 | 2.44 |
|  | Arq Bras Cardiol | 6 | 0.001 | 2.415 |
|  | Korean Circ J | 4 | 0.005 | 2.263 |
|  | Congenit Heart Dis | 2 | 0.014 | 2.259 |
|  | Semin Thorac Cardiovasc Surg | 6 | 0.003 | 2.251 |
|  | Kardiol Pol | 3.5 | 0.003 | 2.234 |
|  | J Cardiopulm Rehabil Prev | 1 | 0.034 | 2.138 |
|  | J Interv Cardiol | 7 | 0.012 | 2.093 |
|  | Cardiology | 5 | 0.006 | 1.998 |
|  | Cardiol J | 2 | 0.01 | 1.977 |
|  | J Interv Card Electrophysiol | 3.5 | 0.008 | 1.96 |
|  | Cardiol Res Pract | 1 | 0.01 | 1.906 |
|  | Cardiovasc J Afr | 3 | 0.006 | 1.879 |
|  | Int Heart J | 1 | 0.007 | 1.731 |
|  | J Invasive Cardiol | 3 | 0.004 | 1.704 |
|  | Scand Cardiovasc J | 7.5 | 0.003 | 1.654 |
|  | Echocardiography | 2 | 0.006 | 1.647 |
|  | Coron Artery Dis | 6.5 | 0.008 | 1.637 |
|  | Ann Noninvasive Electrocardiol | 4 | 0.007 | 1.49 |
|  | J Electrocardiol | 3.5 | 0.008 | 1.47 |
|  | Acta Cardiol | 3.5 | 0.003 | 1.313 |
|  | Tex Heart Inst J | 2 | 0.005 | 1.296 |
|  | Herz | 2 | 0.004 | 1.124 |
|  | Rev Port Cardiol | 3 | 0.002 | 1.061 |
|  | Minerva Cardioangiol | 16 | 0.016 | 0.887 |
| **clinical neurology** | Lancet Neurol | 1 | 0.091 | 41.51 |
|  | Nat Rev Neurol | 1 | 0.017 | 35.2 |
|  | Alzheimers Dement | 1 | 0.016 | 19.962 |
|  | JAMA Neurol | 0 | 0.153 | 16.074 |
|  | Neurology | 1 | 0.033 | 10.663 |
|  | Mov Disord | 1 | 0.02 | 10.414 |
|  | Epilepsy Curr | 0.5 | 0.004 | 8.772 |
|  | Epilepsia | 1 | 0.023 | 7.12 |
|  | Sleep Health | 0 | 0.02 | 6.655 |
|  | Ther Adv Neurol Disord | 3 | 0.05 | 6 |
|  | Parkinsonism Relat Disord | 3 | 0.006 | 5.368 |
|  | J Clin Sleep Med | 1 | 0.049 | 5.255 |
|  | J Neurol | 4 | 0.008 | 5.02 |
|  | Headache | 5 | 0.016 | 4.933 |
|  | Sleep Med | 1 | 0.021 | 4.609 |
|  | Mult Scler Relat Disord | 0 | 0.011 | 4.292 |
|  | Amyotroph Lateral Scler Frontotemporal Degener | 2 | 0.011 | 4.131 |
|  | J Clin Neurol | 3 | 0.006 | 4.089 |
|  | Curr Pain Headache Rep | 5 | 0.022 | 3.731 |
|  | Curr Treat Options Neurol | 1 | 0.019 | 3.617 |
|  | J Pain Res | 1 | 0.018 | 3.507 |
|  | Child Neuropsychol | 2.5 | 0.01 | 3.418 |
|  | Behav Neurol | 1 | 0.016 | 3.29 |
|  | Acta Neurol Scand | 4 | 0.004 | 3.283 |
|  | Semin Neurol | 5 | 0.009 | 3.212 |
|  | Epilepsy Res | 1.5 | 0.005 | 3.143 |
|  | BMC Neurol | 2 | 0.008 | 2.993 |
|  | Pain Res Manag | 4 | 0.02 | 2.987 |
|  | Neurologia | 1.5 | 0.002 | 2.882 |
|  | Can J Neurol Sci | 3 | 0.005 | 2.661 |
|  | Korean J Pain | 1 | 0.027 | 2.538 |
|  | Epileptic Disord | 2 | 0.01 | 2.352 |
|  | Ann Indian Acad Neurol | 0 | 0.007 | 1.626 |
|  | Neurol Neurochir Pol | 1 | 0.013 | 1.386 |
|  | Neurologist | 3 | 0.028 | 1.155 |
|  | Rev Neurol | 9 | 0.001 | 0.756 |
|  | Intensive Care Med | 2 | 0.017 | 19.18 |
|  | Crit Care Med | 2 | 0.02 | 8.416 |
|  | Ann Intensive Care | 4 | 0.016 | 6.068 |
|  | Crit Care Clin | 6 | 0.01 | 4.047 |
|  | J Crit Care | 1 | 0.018 | 3.759 |
|  | Curr Opin Crit Care | 4 | 0.009 | 3.642 |
|  | J Intensive Care Med | 2 | 0.035 | 3.257 |
|  | Crit Care Resusc | 2 | 0.023 | 2.361 |
|  | Med Intensiva | 2 | 0.008 | 2.302 |
|  | Ther Hypothermia Temp Manag | 1 | 0.054 | 1.474 |
| **dermatology** | JAMA Dermatol | 1 | 0.102 | 10.725 |
|  | J Am Acad Dermatol | 1 | 0.029 | 10.238 |
|  | J Invest Dermatol | 1.5 | 0.006 | 8.452 |
|  | Br J Dermatol | 1 | 0.018 | 8.407 |
|  | Am J Clin Dermatol | 2 | 0.026 | 6.39 |
|  | J Eur Acad Dermatol Venereol | 1 | 0.02 | 5.977 |
|  | J Dermatol Sci | 1 | 0.006 | 5.052 |
|  | J Dtsch Dermatol Ges | 3 | 0.012 | 4.796 |
|  | Acta Derm Venereol | 4.5 | 0.007 | 4.379 |
|  | Dermatol Clin | 2 | 0.011 | 4.246 |
|  | Dermatitis | 1.5 | 0.017 | 3.943 |
|  | Dermatology | 4 | 0.014 | 3.888 |
|  | Eur J Dermatol | 4 | 0.011 | 3.778 |
|  | J Dermatol | 2 | 0.007 | 3.626 |
|  | Exp Dermatol | 3 | 0.006 | 3.489 |
|  | Clin Dermatol | 2 | 0.01 | 3.392 |
|  | Indian J Dermatol Venereol Leprol | 2 | 0.005 | 3.251 |
|  | Photodermatol Photoimmunol Photomed | 5.5 | 0.014 | 3.162 |
|  | J Dermatolog Treat | 1 | 0.016 | 3.062 |
|  | Dermatol Ther | 1 | 0.019 | 2.912 |
|  | J Cosmet Dermatol | 4 | 0.008 | 2.89 |
|  | Clin Exp Dermatol | 4.5 | 0.009 | 2.661 |
|  | J Wound Care | 3 | 0.003 | 2.63 |
|  | Int J Dermatol | 3 | 0.007 | 2.48 |
|  | Australas J Dermatol | 3.5 | 0.008 | 2.306 |
|  | Indian J Dermatol | 2 | 0.006 | 2.195 |
|  | Ann Dermatol | 2.5 | 0.012 | 2.095 |
|  | An Bras Dermatol | 2.5 | 0.009 | 2.057 |
|  | J Cutan Med Surg | 4 | 0.011 | 2.029 |
|  | J Drugs Dermatol | 2 | 0.017 | 1.92 |
|  | G Ital Dermatol Venereol | 2 | 0.008 | 1.671 |
|  | Am J Dermatopathol | 1 | 0.008 | 1.435 |
|  | Acta Dermatovenerol Croat | 6 | 0.014 | 1.429 |
|  | Ann Dermatol Venereol | 2 | 0.002 | 0.82 |
|  | Hautarzt | 10.5 | 0.001 | 0.699 |
| **emergency medicine** | Ann Emerg Med | 1 | 0.02 | 6.107 |
|  | Acad Emerg Med | 2 | 0.019 | 3.891 |
|  | Scand J Trauma Resusc Emerg Med | 1 | 0.009 | 3.263 |
|  | Emerg Med J | 3 | 0.009 | 3.135 |
|  | Eur J Trauma Emerg Surg | 1 | 0.016 | 3.088 |
|  | West J Emerg Med | 2 | 0.014 | 2.788 |
|  | BMC Emerg Med | 3 | 0.058 | 2.54 |
|  | Emerg Med Clin North Am | 2.5 | 0.011 | 2.346 |
|  | CJEM | 1 | 0.014 | 2.257 |
|  | Eur J Emerg Med | 6 | 0.005 | 2.155 |
|  | Am J Emerg Med | 2 | 0.01 | 2.101 |
|  | Emerg Med Australas | 4.5 | 0.01 | 2.064 |
|  | World J Emerg Med | 5 | 0.018 | 2.04 |
|  | Prehosp Disaster Med | 0.5 | 0.006 | 1.928 |
|  | J Emerg Med | 1 | 0.013 | 1.584 |
| **endocrinology & metabolism** | Nat Rev Endocrinol | 1 | 0.047 | 36.757 |
|  | Lancet Diabetes Endocrinol | 1 | 0.041 | 29.79 |
|  | Endocr Rev | 5 | 0.035 | 24.715 |
|  | Diabetes Care | 2 | 0.021 | 17.067 |
|  | Trends Endocrinol Metab | 1.5 | 0.009 | 13.851 |
|  | Obes Rev | 3 | 0.009 | 12.125 |
|  | Diabetologia | 4 | 0.005 | 9.097 |
|  | Diabetes | 8 | 0.004 | 9.091 |
|  | Thyroid | 1 | 0.032 | 9.034 |
|  | Rev Endocr Metab Disord | 1 | 0.023 | 8.264 |
|  | Metabolism | 3 | 0.003 | 7.867 |
|  | Mol Metab | 1 | 0.009 | 7.864 |
|  | World J Diabetes | 1 | 0.007 | 7.348 |
|  | J Bone Miner Res | 2 | 0.02 | 6.893 |
|  | J Clin Endocrinol Metab | 3 | 0.027 | 6.792 |
|  | Diabetes Obes Metab | 2 | 0.022 | 6.587 |
|  | Eur J Endocrinol | 2 | 0.023 | 6.341 |
|  | Curr Osteoporos Rep | 2 | 0.019 | 6.064 |
|  | Diabetes Metab Res Rev | 3 | 0.008 | 5.324 |
|  | Best Pract Res Clin Endocrinol Metab | 4 | 0.026 | 5.26 |
|  | Curr Diab Rep | 1 | 0.01 | 5.221 |
|  | Bone | 5 | 0.008 | 5.175 |
|  | J Endocrinol | 5 | 0.003 | 4.971 |
|  | Diabetes Metab | 7 | 0.007 | 4.931 |
|  | Diabetes Res Clin Pract | 2 | 0.004 | 4.876 |
|  | Diabetes Metab J | 1 | 0.014 | 4.865 |
|  | Endocrinology | 6.5 | 0.001 | 4.809 |
|  | Osteoporos Int | 1 | 0.011 | 4.802 |
|  | Endocrinol Metab Clin North Am | 5.5 | 0.015 | 4.755 |
|  | J Mol Endocrinol | 2 | 0.007 | 4.755 |
|  | Diabetes Technol Ther | 2 | 0.01 | 4.699 |
|  | Curr Opin Endocrinol Diabetes Obes | 1 | 0.016 | 4.538 |
|  | Calcif Tissue Int | 4.5 | 0.003 | 4.306 |
|  | Diabet Med | 3 | 0.009 | 4.288 |
|  | Endocr Pract | 3 | 0.013 | 4.205 |
|  | Pituitary | 1 | 0.02 | 4.167 |
|  | BMJ Open Diabetes Res Care | 1.5 | 0.013 | 3.98 |
|  | J Endocrinol Invest | 5.5 | 0.005 | 3.881 |
|  | J Diabetes | 4 | 0.024 | 3.853 |
|  | Acta Diabetol | 2 | 0.004 | 3.77 |
|  | Endocrine | 1 | 0.007 | 3.613 |
|  | Diabetes Metab Syndr Obes | 3 | 0.02 | 3.602 |
|  | Diabetol Metab Syndr | 2 | 0.007 | 3.426 |
|  | J Diabetes Complications | 1.5 | 0.006 | 3.378 |
|  | Diabetes Ther | 1 | 0.019 | 3.261 |
|  | Int J Endocrinol | 3 | 0.004 | 3.167 |
|  | J Clin Densitom | 1 | 0.013 | 3.165 |
|  | Arch Endocrinol Metab | 1 | 0.013 | 3.105 |
|  | BMC Endocr Disord | 4 | 0.017 | 2.879 |
|  | Horm Metab Res | 1 | 0.003 | 2.772 |
|  | Endocr J | 1 | 0.006 | 2.404 |
|  | Exp Clin Endocrinol Diabetes | 9 | 0.008 | 2.376 |
|  | Minerva Endocrinol | 3 | 0.006 | 1.924 |
|  | Endocr Res | 8.5 | 0.009 | 1.876 |
| **gastroenterology & hepatology** | Nat Rev Gastroenterol Hepatol | 2 | 0.024 | 38.143 |
|  | Gastroenterology | 2 | 0.024 | 23.936 |
|  | Gut | 2 | 0.018 | 22.223 |
|  | J Hepatol | 1 | 0.022 | 20.888 |
|  | Hepatology | 1 | 0.023 | 16.555 |
|  | Am J Gastroenterol | 2 | 0.024 | 12.591 |
|  | Clin Gastroenterol Hepatol | 1 | 0.074 | 10.03 |
|  | J Crohns Colitis | 2 | 0.018 | 9.485 |
|  | Gastrointest Endosc | 1 | 0.028 | 8.955 |
|  | Cell Mol Gastroenterol Hepatol | 2 | 0.042 | 8.69 |
|  | J Gastroenterol | 3 | 0.007 | 6.769 |
|  | Clin Liver Dis | 2 | 0.038 | 6.603 |
|  | Semin Liver Dis | 3 | 0.018 | 6.503 |
|  | Hepatol Int | 1 | 0.008 | 5.975 |
|  | Clin Transl Gastroenterol | 1 | 0.021 | 5.551 |
|  | Inflamm Bowel Dis | 2 | 0.024 | 5.328 |
|  | Liver Int | 3 | 0.012 | 5.276 |
|  | World J Gastroenterol | 3 | 0.007 | 5.044 |
|  | Gastroenterol Clin North Am | 6 | 0.009 | 4.805 |
|  | Clin Mol Hepatol | 2 | 0.02 | 4.793 |
|  | United European Gastroenterol J | 0 | 0.006 | 4.687 |
|  | J Clin Transl Hepatol | 1 | 0.024 | 4.608 |
|  | Best Pract Res Clin Gastroenterol | 8 | 0.007 | 4.438 |
|  | Therap Adv Gastroenterol | 1.5 | 0.032 | 4.333 |
|  | Pancreatology | 1.5 | 0.008 | 4.262 |
|  | J Gastroenterol Hepatol | 3 | 0.008 | 4.144 |
|  | Curr Opin Gastroenterol | 3 | 0.011 | 3.997 |
|  | Gut Liver | 1 | 0.012 | 3.989 |
|  | Dig Liver Dis | 2 | 0.009 | 3.947 |
|  | Endosc Ultrasound | 1 | 0.009 | 3.84 |
|  | Expert Rev Gastroenterol Hepatol | 2 | 0.015 | 3.785 |
|  | Hepatol Res | 2 | 0.006 | 3.651 |
|  | BMC Gastroenterol | 2 | 0.018 | 3.441 |
|  | Pancreas | 1 | 0.012 | 3.417 |
|  | Dig Dis Sci | 2 | 0.011 | 3.392 |
|  | Dis Esophagus | 1 | 0.021 | 3.343 |
|  | J Clin Gastroenterol | 2.5 | 0.009 | 3.308 |
|  | Can J Gastroenterol Hepatol | 1.5 | 0.007 | 3.149 |
|  | Dig Dis | 2 | 0.005 | 3.098 |
|  | Digestion | 2 | 0.006 | 3.062 |
|  | Clin Res Hepatol Gastroenterol | 1 | 0.005 | 2.954 |
|  | Scand J Gastroenterol | 4 | 0.009 | 2.801 |
|  | Eur J Gastroenterol Hepatol | 4 | 0.009 | 2.613 |
|  | Saudi J Gastroenterol | 1 | 0.007 | 2.46 |
|  | Ann Hepatol | 3 | 0.011 | 2.449 |
|  | Rev Esp Enferm Dig | 1 | 0.003 | 2.328 |
|  | Gastroenterol Res Pract | 2 | 0.008 | 2.279 |
|  | J Gastrointestin Liver Dis | 2 | 0.01 | 2.258 |
|  | J Dig Dis | 1.5 | 0.012 | 2.174 |
|  | Minerva Gastroenterol Dietol | 8 | 0.024 | 2.111 |
|  | Z Gastroenterol | 3 | 0.009 | 1.759 |
|  | Gastroenterol Hepatol | 7 | 0.007 | 1.738 |
|  | Turk J Gastroenterol | 4.5 | 0.002 | 1.707 |
|  | Arab J Gastroenterol | 3 | 0.017 | 1.541 |
|  | Hepat Mon | 1 | 0.004 | 1.409 |
|  | Acta Gastroenterol Belg | 5 | 0.013 | 1.022 |
| **geriatrics & gerontology** | Age Ageing | 3.5 | 0.008 | 8.222 |
|  | J Am Med Dir Assoc | 4 | 0.009 | 6.462 |
|  | Gerontology | 6 | 0.004 | 5.685 |
|  | Biogerontology | 2 | 0.015 | 5.252 |
|  | Clin Interv Aging | 1.5 | 0.006 | 4.744 |
|  | Rejuvenation Res | 3 | 0.012 | 4.342 |
|  | Exp Gerontol | 5 | 0.002 | 4.297 |
|  | Arch Gerontol Geriatr | 3.5 | 0.005 | 3.734 |
|  | Clin Geriatr Med | 3 | 0.067 | 3.472 |
|  | Aging Clin Exp Res | 8 | 0.003 | 3.428 |
| **hematology** | Blood | 1 | 0.034 | 19.676 |
|  | Lancet Haematol | 1 | 0.082 | 16.564 |
|  | Haematologica | 2 | 0.022 | 9.754 |
|  | Am J Hematol | 1 | 0.029 | 7.954 |
|  | Blood Rev | 3.5 | 0.042 | 7.773 |
|  | Blood Adv | 0 | 0.004 | 6.795 |
|  | Br J Haematol | 1 | 0.019 | 6.455 |
|  | Transfus Med Rev | 2 | 0.026 | 5.063 |
|  | Semin Hematol | 6 | 0.009 | 4.132 |
|  | Haemophilia | 2.5 | 0.014 | 4.011 |
|  | Curr Opin Hematol | 1 | 0.019 | 3.74 |
|  | Ann Hematol | 2 | 0.011 | 3.541 |
|  | Blood Transfus | 2.5 | 0.01 | 3.47 |
|  | Transfusion | 1 | 0.017 | 3.178 |
|  | Int J Lab Hematol | 1 | 0.017 | 2.921 |
|  | Eur J Haematol | 2 | 0.009 | 2.817 |
|  | Best Pract Res Clin Haematol | 5 | 0.01 | 2.808 |
|  | Expert Rev Hematol | 3 | 0.022 | 2.777 |
|  | J Clin Apher | 3.5 | 0.015 | 2.675 |
|  | Blood Cells Mol Dis | 1 | 0.013 | 2.508 |
|  | Int J Hematol | 4 | 0.003 | 2.378 |
|  | Vox Sang | 3.5 | 0.004 | 2.181 |
|  | Transfus Med | 6 | 0.008 | 2.059 |
|  | Hematology | 2 | 0.005 | 2.033 |
|  | Transfus Apher Sci | 1 | 0.007 | 1.781 |
|  | Acta Haematol | 3 | 0.005 | 1.767 |
|  | Hamostaseologie | 2 | 0.011 | 1.495 |
|  | Blood Coagul Fibrinolysis | 4.5 | 0.007 | 1.328 |
| **infectious diseases** | Lancet Infect Dis | 1 | 0.07 | 25.023 |
|  | Euro Surveill | 1 | 0.012 | 6.015 |
|  | J Infect | 3 | 0.013 | 5.427 |
|  | Infect Dis Ther | 0 | 0.026 | 4.798 |
|  | Curr Opin Infect Dis | 2 | 0.026 | 4.729 |
|  | Int J Infect Dis | 4 | 0.014 | 4.01 |
|  | Sex Transm Infect | 2.5 | 0.009 | 3.783 |
|  | Infection | 2 | 0.008 | 3.489 |
|  | BMC Infect Dis | 1 | 0.012 | 3.401 |
|  | Curr Infect Dis Rep | 1 | 0.009 | 3.388 |
|  | HIV Med | 3 | 0.014 | 3.317 |
|  | Infect Genet Evol | 2 | 0.003 | 3.188 |
|  | AIDS Res Ther | 2 | 0.01 | 2.782 |
|  | Sex Transm Dis | 3 | 0.012 | 2.692 |
|  | Braz J Infect Dis | 3.5 | 0.004 | 2.347 |
|  | Med Mal Infect | 1 | 0.004 | 1.858 |
|  | J Infect Dev Ctries | 3 | 0.005 | 1.484 |
|  | Jpn J Infect Dis | 5 | 0.004 | 1.297 |
|  | Rev Chilena Infectol | 4 | 0.003 | 0.585 |
| **medicine, general & internal** | N Engl J Med | 1 | 0.038 | 89.666 |
|  | Lancet | 1 | 0.011 | 77.237 |
|  | JAMA | 1 | 0.021 | 60.145 |
|  | BMJ | 1 | 0.017 | 38.657 |
|  | Ann Intern Med | 1 | 0.03 | 25.267 |
|  | JAMA Intern Med | 0 | 0.122 | 23.067 |
|  | PLoS Med | 1 | 0.03 | 14.412 |
|  | BMC Med | 1 | 0.022 | 10.249 |
|  | CMAJ | 1 | 0.013 | 10.033 |
|  | Cochrane Database Syst Rev | 1 | 0.111 | 9.871 |
|  | Mayo Clin Proc | 2 | 0.022 | 9.422 |
|  | J Intern Med | 3 | 0.012 | 8.302 |
|  | Med J Aust | 4.5 | 0.001 | 6.905 |
|  | Dtsch Arztebl Int | 1 | 0.017 | 6.459 |
|  | Am J Med | 2 | 0.015 | 6.011 |
|  | Br Med Bull | 5 | 0.005 | 5.044 |
|  | Syst Rev | 2.5 | 0.013 | 5.038 |
|  | Med Clin North Am | 2 | 0.005 | 4.828 |
|  | J R Soc Med | 20 | 0.001 | 4.691 |
|  | Eur J Intern Med | 2 | 0.007 | 4.567 |
|  | J Clin Med | 1 | 0.021 | 4.566 |
|  | Ann Med | 3 | 0.003 | 4.554 |
|  | Int J Med Sci | 3 | 0.006 | 3.508 |
|  | QJM | 1 | 0.019 | 3.452 |
|  | BMJ Open | 1 | 0.007 | 3.424 |
|  | Panminerva Med | 11.5 | 0.021 | 3.352 |
|  | J Hosp Med | 1 | 0.02 | 3.299 |
|  | Mil Med Res | 0 | 0.027 | 3.285 |
|  | Arch Med Sci | 3 | 0.006 | 3.257 |
|  | Korean J Intern Med | 4 | 0.008 | 3.225 |
|  | Intern Emerg Med | 1 | 0.006 | 3.168 |
|  | J Formos Med Assoc | 2 | 0.003 | 3.16 |
|  | Ups J Med Sci | 4 | 0.008 | 3.08 |
|  | Postgrad Med | 3 | 0.004 | 2.852 |
|  | Postgrad Med J | 7 | 0.003 | 2.821 |
|  | Int J Clin Pract | 3.5 | 0.007 | 2.726 |
|  | Int J Gen Med | 4 | 0.008 | 2.724 |
|  | Am J Med Sci | 5 | 0.005 | 2.71 |
|  | J Korean Med Sci | 3 | 0.004 | 2.467 |
|  | J Chin Med Assoc | 5 | 0.003 | 2.452 |
|  | Yonsei Med J | 8 | 0.003 | 2.398 |
|  | Med Princ Pract | 0 | 0.009 | 2.388 |
|  | Cleve Clin J Med | 4 | 0.007 | 2.325 |
|  | Hong Kong Med J | 4 | 0.007 | 2.319 |
|  | Swiss Med Wkly | 5.5 | 0.007 | 2.296 |
|  | J Res Med Sci | 2 | 0.002 | 2.25 |
|  | Intern Med J | 1 | 0.009 | 2.089 |
|  | S Afr Med J | 6 | 0.001 | 2.068 |
|  | Singapore Med J | 3 | 0.002 | 2.053 |
|  | Saudi Med J | 9 | 0.002 | 2.051 |
|  | Ann Saudi Med | 5 | 0.01 | 2.044 |
|  | J Postgrad Med | 3.5 | 0.005 | 1.92 |
|  | Arch Iran Med | 2 | 0.014 | 1.777 |
|  | World J Clin Cases | 1 | 0.01 | 1.742 |
|  | Croat Med J | 6.5 | 0.006 | 1.696 |
|  | Rev Invest Clin | 7 | 0.018 | 1.696 |
|  | J Natl Med Assoc | 9.5 | 0.002 | 1.668 |
|  | Wien Klin Wochenschr | 5 | 0.003 | 1.635 |
|  | Neth J Med | 5.5 | 0.005 | 1.556 |
|  | Mil Med | 3 | 0.003 | 1.47 |
|  | Afr Health Sci | 5.5 | 0.005 | 1.442 |
|  | Ir J Med Sci | 1 | 0.003 | 1.441 |
|  | Sao Paulo Med J | 3 | 0.007 | 1.415 |
|  | Bratisl Lek Listy | 7.5 | 0.002 | 1.365 |
|  | Dan Med J | 1.5 | 0.007 | 1.357 |
|  | Pak J Med Sci | 0 | 0.003 | 1.276 |
|  | Rev Clin Esp | 1 | 0.01 | 1.221 |
|  | Intern Med | 3 | 0.006 | 1.22 |
|  | South Med J | 15 | 0.003 | 1.153 |
|  | Iran Red Crescent Med J | 2 | 0.004 | 1.152 |
|  | J Nippon Med Sch | 6.5 | 0.007 | 1.112 |
|  | Acta Clin Belg | 2 | 0.014 | 1.102 |
|  | Turk J Med Sci | 1 | 0.004 | 1.063 |
|  | Natl Med J India | 18 | 0.011 | 0.918 |
|  | Rev Med Interne | 4 | 0.003 | 0.909 |
|  | Scott Med J | 22 | 0.001 | 0.849 |
|  | JAAPA | 5 | 0.002 | 0.835 |
|  | Acta Clin Croat | 3 | 0.015 | 0.766 |
|  | J Coll Physicians Surg Pak | 2.5 | 0.006 | 0.735 |
|  | Rev Med Chil | 9 | 0.005 | 0.725 |
|  | Dtsch Med Wochenschr | 7 | 0 | 0.557 |
|  | Orv Hetil | 4.5 | 0.001 | 0.461 |
|  | West Indian Med J | 3 | 0.005 | 0.381 |
|  | Vojnosanit Pregl | 4 | 0.007 | 0.378 |
|  | Srp Arh Celok Lek | 2 | 0.007 | 0.358 |
| **oncology** | CA Cancer J Clin | 1 | 0.074 | 332.984 |
|  | Nat Rev Cancer | 2.5 | 0.009 | 68.839 |
|  | Nat Rev Clin Oncol | 1 | 0.029 | 52.441 |
|  | Lancet Oncol | 1 | 0.14 | 44.11 |
|  | Cancer Discov | 1 | 0.015 | 39.115 |
|  | J Clin Oncol | 1 | 0.071 | 33.883 |
|  | JAMA Oncol | 0 | 0.105 | 29.807 |
|  | Ann Oncol | 1 | 0.034 | 22.845 |
|  | Semin Cancer Biol | 2 | 0.008 | 14.564 |
|  | J Natl Cancer Inst | 1 | 0.016 | 13.893 |
|  | Cancer Res | 5 | 0.002 | 12.843 |
|  | Clin Cancer Res | 1 | 0.009 | 12.836 |
|  | Cancer Treat Rev | 2.5 | 0.016 | 11.143 |
|  | Cancer Metastasis Rev | 8 | 0.029 | 10.016 |
|  | J Exp Clin Cancer Res | 5 | 0.003 | 9.976 |
|  | J Natl Compr Canc Netw | 2 | 0.022 | 9.856 |
|  | Cancer Lett | 6 | 0 | 8.64 |
|  | Int J Cancer | 2 | 0.008 | 7.968 |
|  | Cancer | 2 | 0.012 | 7.921 |
|  | Br J Cancer | 1 | 0.008 | 7.57 |
|  | Breast Cancer Res | 4 | 0.007 | 7.426 |
|  | Mol Oncol | 8 | 0.04 | 7.405 |
|  | Mol Cancer Ther | 5 | 0.002 | 7.019 |
|  | Cancer Sci | 3.5 | 0.002 | 6.33 |
|  | Front Oncol | 4 | 0.003 | 6.264 |
|  | Am J Cancer Res | 4 | 0.024 | 5.863 |
|  | Semin Oncol | 3 | 0.002 | 5.47 |
|  | Carcinogenesis | 7 | 0.001 | 5.41 |
|  | Int J Oncol | 7 | 0 | 4.79 |
|  | Breast Cancer Res Treat | 2 | 0.005 | 4.698 |
|  | Curr Treat Options Oncol | 2 | 0.007 | 4.66 |
|  | Curr Oncol Rep | 1.5 | 0.027 | 4.617 |
|  | Cancer Med | 2 | 0.008 | 4.609 |
|  | Clin Colorectal Cancer | 1 | 0.036 | 4.538 |
|  | J Oncol | 3 | 0.009 | 4.499 |
|  | Clin Lung Cancer | 1 | 0.01 | 4.417 |
|  | BMC Cancer | 4 | 0.003 | 4.372 |
|  | Target Oncol | 2.5 | 0.029 | 4.369 |
|  | J Oncol Pract | 1.5 | 0.008 | 4.347 |
|  | Cancer Biol Ther | 4 | 0.002 | 4.342 |
|  | J Cancer | 3 | 0.004 | 4.277 |
|  | Acta Oncol | 3 | 0.005 | 4.236 |
|  | J Cancer Res Clin Oncol | 1 | 0.007 | 4.201 |
|  | Cancer J | 3 | 0.013 | 4.013 |
|  | Oncol Rep | 4 | 0.001 | 4.011 |
|  | Cancer Manag Res | 2.5 | 0.015 | 3.947 |
|  | Curr Opin Oncol | 3 | 0.009 | 3.705 |
|  | Expert Rev Anticancer Ther | 2 | 0.007 | 3.665 |
|  | Curr Probl Cancer | 5 | 0.048 | 3.615 |
|  | Photodiagnosis Photodyn Ther | 3 | 0.011 | 3.603 |
|  | Cancer Control | 4 | 0.011 | 3.429 |
|  | Clin Breast Cancer | 2 | 0.01 | 3.356 |
|  | Med Oncol | 1.5 | 0.004 | 3.304 |
|  | Int J Clin Oncol | 1 | 0.006 | 3.238 |
|  | Curr Oncol | 2 | 0.007 | 3.089 |
|  | Clin Transl Oncol | 5.5 | 0.003 | 3.057 |
|  | Future Oncol | 1.5 | 0.005 | 3.009 |
|  | Oncology | 3 | 0.011 | 3.006 |
|  | Jpn J Clin Oncol | 3 | 0.006 | 2.847 |
|  | Am J Clin Oncol | 1 | 0.013 | 2.831 |
|  | Eur J Cancer Prev | 3 | 0.006 | 2.83 |
|  | J Adolesc Young Adult Oncol | 2 | 0.048 | 2.796 |
|  | Asia Pac J Clin Oncol | 5 | 0.009 | 2.627 |
|  | Hered Cancer Clin Pract | 4 | 0.1 | 2.627 |
|  | Oncol Lett | 1 | 0.001 | 2.575 |
|  | Anticancer Res | 4 | 0.001 | 2.418 |
|  | Cancer Invest | 5 | 0.005 | 2.344 |
|  | Neoplasma | 5.5 | 0.002 | 2.337 |
|  | J BUON | 4 | 0.005 | 1.987 |
|  | Tumori | 11 | 0.002 | 1.722 |
|  | J Cancer Res Ther | 4 | 0.003 | 1.617 |
|  | Indian J Cancer | 13.5 | 0.018 | 1.306 |
|  | Bull Cancer | 3 | 0.006 | 1.025 |
| **pathology** | Annu Rev Pathol | 1 | 0.024 | 25.061 |
|  | Mod Pathol | 3 | 0.01 | 8.048 |
|  | J Mol Diagn | 1 | 0.014 | 6.343 |
|  | Am J Pathol | 8 | 0.002 | 5.138 |
|  | Pathology | 2 | 0.007 | 4.715 |
|  | Adv Anat Pathol | 1 | 0.014 | 4.46 |
|  | Expert Rev Mol Diagn | 1 | 0.017 | 4.419 |
|  | Virchows Arch | 2 | 0.006 | 3.619 |
|  | Hum Pathol | 5 | 0.006 | 3.456 |
|  | J Clin Pathol | 9 | 0.003 | 3.12 |
|  | Exp Mol Pathol | 3 | 0.005 | 2.935 |
|  | Am J Clin Pathol | 5 | 0.005 | 2.775 |
|  | Diagn Pathol | 2 | 0.005 | 2.759 |
|  | Pathol Res Pract | 3 | 0.001 | 2.74 |
|  | Pathol Int | 4 | 0.005 | 2.671 |
|  | Int J Exp Pathol | 7 | 0.016 | 2.447 |
|  | Acta Cytol | 12 | 0.004 | 2.324 |
|  | Ann Diagn Pathol | 17 | 0.003 | 2.276 |
|  | Cytojournal | 1 | 0.018 | 1.629 |
|  | Pol J Pathol | 6 | 0.013 | 1.074 |
|  | Indian J Pathol Microbiol | 4 | 0.001 | 0.815 |
|  | JAMA Pediatr | 0 | 0.13 | 16.709 |
|  | Pediatrics | 1 | 0.039 | 8.109 |
|  | Arch Dis Child Fetal Neonatal Ed | 1 | 0.046 | 5.191 |
|  | Semin Fetal Neonatal Med | 2 | 0.038 | 5.067 |
|  | J Pediatr | 1 | 0.024 | 5.042 |
|  | Pediatr Obes | 0 | 0.02 | 4.12 |
|  | Arch Dis Child | 1 | 0.011 | 3.96 |
|  | Acad Pediatr | 2 | 0.01 | 3.955 |
|  | Pediatr Res | 2 | 0.008 | 3.926 |
|  | Pediatr Clin North Am | 3 | 0.004 | 3.866 |
|  | Neonatology | 2 | 0.026 | 3.774 |
|  | Front Pediatr | 2 | 0.004 | 3.607 |
|  | Child Obes | 2.5 | 0.007 | 3.463 |
|  | Curr Opin Pediatr | 2 | 0.021 | 3.235 |
|  | Eur J Pediatr | 1 | 0.012 | 3.152 |
|  | BMC Pediatr | 1 | 0.016 | 2.782 |
|  | Ital J Pediatr | 2 | 0.015 | 2.672 |
|  | Paediatr Child Health | 5.5 | 0.005 | 2.483 |
|  | Pediatr Neonatol | 1.5 | 0.014 | 2.174 |
|  | Paediatr Int Child Health | 0 | 0.015 | 2.145 |
|  | J Paediatr Child Health | 4 | 0.009 | 2.137 |
|  | Arch Dis Child Educ Pract Ed | 1 | 0.016 | 2.005 |
|  | Indian J Pediatr | 4 | 0.001 | 1.868 |
|  | Indian Pediatr | 6 | 0.002 | 1.709 |
|  | Pediatr Int | 4 | 0.004 | 1.439 |
|  | Pediatr Ann | 3 | 0.008 | 1.404 |
|  | Minerva Pediatr | 2 | 0.014 | 1.198 |
|  | Klin Padiatr | 1.5 | 0.002 | 1.176 |
|  | Arch Argent Pediatr | 0 | 0.006 | 0.708 |
|  | Turk J Pediatr | 7 | 0.003 | 0.642 |
| **radiology, nuclear medicine & medical imaging** | Radiology | 3 | 0.011 | 10.389 |
|  | J Nucl Med | 5.5 | 0.003 | 8.573 |
|  | Eur J Nucl Med Mol Imaging | 4 | 0.007 | 8.391 |
|  | Radiographics | 2 | 0.02 | 7.269 |
|  | Clin Nucl Med | 7 | 0.003 | 6.918 |
|  | Invest Radiol | 9 | 0.001 | 5.958 |
|  | J Am Coll Radiol | 2 | 0.004 | 4.91 |
|  | Insights Imaging | 1 | 0.014 | 4.879 |
|  | Eur Radiol | 3 | 0.007 | 4.87 |
|  | J Magn Reson Imaging | 5 | 0.002 | 4.642 |
|  | Magn Reson Med | 3 | 0.003 | 4.616 |
|  | Semin Nucl Med | 5.5 | 0.008 | 4.392 |
|  | AJR Am J Roentgenol | 2 | 0.012 | 4.073 |
|  | Korean J Radiol | 4 | 0.01 | 3.947 |
|  | Med Phys | 2.5 | 0 | 3.767 |
|  | Eur J Radiol | 3 | 0.008 | 3.539 |
|  | Mol Imaging Biol | 3 | 0.008 | 3.389 |
|  | Diagn Interv Imaging | 1 | 0.008 | 3.206 |
|  | Br J Radiol | 4.5 | 0.003 | 3.016 |
|  | Acad Radiol | 3 | 0.007 | 2.751 |
|  | BMC Med Imaging | 2 | 0.023 | 2.683 |
|  | Radiol Med | 5 | 0.006 | 2.624 |
|  | Magn Reson Imaging | 3.5 | 0.001 | 2.608 |
|  | Clin Radiol | 6 | 0.006 | 2.597 |
|  | Diagn Interv Radiol | 1.5 | 0.016 | 2.591 |
|  | J Thorac Imaging | 9 | 0.01 | 2.463 |
|  | Magn Reson Imaging Clin N Am | 4 | 0.004 | 2.255 |
|  | Radiol Clin North Am | 4.5 | 0.007 | 2.172 |
|  | Rofo | 8 | 0.006 | 2.152 |
|  | Ann Nucl Med | 6 | 0.005 | 2.151 |
|  | Q J Nucl Med Mol Imaging | 1 | 0.017 | 2.109 |
|  | Semin Intervent Radiol | 2.5 | 0.013 | 2.083 |
|  | Acta Radiol | 3 | 0.004 | 1.92 |
|  | Semin Ultrasound CT MR | 3 | 0.011 | 1.884 |
|  | Can Assoc Radiol J | 10 | 0.003 | 1.869 |
|  | Jpn J Radiol | 3 | 0.008 | 1.857 |
|  | Semin Musculoskelet Radiol | 8 | 0.009 | 1.84 |
|  | J Med Imaging Radiat Oncol | 4 | 0.009 | 1.645 |
|  | Nucl Med Commun | 5 | 0.006 | 1.631 |
|  | J Comput Assist Tomogr | 4 | 0.003 | 1.592 |
|  | Clin Imaging | 3 | 0.004 | 1.481 |
|  | Ultrasound Q | 2 | 0.015 | 1.444 |
|  | Semin Roentgenol | 7 | 0.003 | 0.715 |
|  | Iran J Radiol | 1 | 0.011 | 0.534 |
| **urology & nephrology** | Nat Rev Nephrol | 2 | 0.044 | 26.996 |
|  | Eur Urol | 1 | 0.022 | 21.259 |
|  | Nat Rev Urol | 2 | 0.009 | 12.574 |
|  | J Am Soc Nephrol | 2 | 0.045 | 10.578 |
|  | Kidney Int | 2 | 0.021 | 10.558 |
|  | Clin J Am Soc Nephrol | 1 | 0.097 | 8.427 |
|  | Am J Kidney Dis | 3 | 0.032 | 8.418 |
|  | J Urol | 2 | 0.008 | 6.413 |
|  | BJU Int | 1.5 | 0.009 | 5.225 |
|  | Semin Nephrol | 4 | 0.014 | 5.061 |
|  | J Sex Med | 3 | 0.01 | 4.699 |
|  | Kidney Int Rep | 0 | 0.007 | 4.196 |
|  | Adv Chronic Kidney Dis | 4 | 0.018 | 4.134 |
|  | Clin Kidney J | 2 | 0.033 | 3.96 |
|  | Am J Nephrol | 4 | 0.01 | 3.953 |
|  | World J Urol | 2 | 0.006 | 3.874 |
|  | J Nephrol | 4 | 0.01 | 3.667 |
|  | J Endourol | 2 | 0.009 | 3.089 |
|  | Urolithiasis | 2 | 0.007 | 3.011 |
|  | Ther Adv Urol | 3 | 0.038 | 3.01 |
|  | Int J Urol | 0 | 0.006 | 2.986 |
|  | Neurourol Urodyn | 3 | 0.007 | 2.898 |
|  | BMC Nephrol | 3 | 0.01 | 2.879 |
|  | Curr Urol Rep | 2 | 0.01 | 2.763 |
|  | Semin Dial | 3 | 0.022 | 2.668 |
|  | Urol Clin North Am | 2 | 0.007 | 2.648 |
|  | Clin Exp Nephrol | 3 | 0.008 | 2.613 |
|  | Nephron | 22 | 0.004 | 2.586 |
|  | Nephron Clin Pract | 3.5 | 0.018 | 2.586 |
|  | Nephron Physiol | 5 | 0.019 | 2.586 |
|  | Urology | 1 | 0.008 | 2.564 |
|  | Int J Impot Res | 10 | 0.002 | 2.433 |
|  | BMC Urol | 3 | 0.009 | 2.399 |
|  | Ren Fail | 3 | 0.006 | 2.364 |
|  | Curr Opin Urol | 1 | 0.01 | 2.271 |
|  | Prostate Int | 1 | 0.033 | 2.248 |
|  | Nefrologia | 1 | 0.006 | 2.217 |
|  | Int Urol Nephrol | 2 | 0.004 | 2.199 |
|  | Perit Dial Int | 4 | 0.009 | 2.161 |
|  | Urol Int | 2 | 0.013 | 1.91 |
|  | Hemodial Int | 3 | 0.022 | 1.879 |
|  | Scand J Urol | 1 | 0.012 | 1.856 |
|  | Can Urol Assoc J | 3 | 0.004 | 1.729 |
|  | Urol J | 3 | 0.014 | 1.641 |
|  | Contrib Nephrol | 2 | 0.002 | 1.609 |
|  | Can J Urol | 11 | 0.021 | 1.571 |
|  | Clin Nephrol | 11 | 0.008 | 1.224 |
|  | Nephrol Ther | 4 | 0.007 | 0.792 |
|  | Prog Urol | 4 | 0.005 | 0.744 |
|  | Urologe A | 16 | 0.012 | 0.566 |
|  | Arch Esp Urol | 3 | 0.008 | 0.498 |
